# Performance of free-flow field-step electrophoresis as cleanup step for the non-target analysis of environmental water samples

**DOI:** 10.1007/s00216-021-03856-w

**Published:** 2022-01-31

**Authors:** Tobias Rösch, Gerhard Weber, Tobias Bader, Anna-Jorina Wicht, Carolin Huhn

**Affiliations:** 1 Institute of Physical and Theoretical Chemistry, University of Tübingen, Auf der Morgenstelle 18, Tübingen, Germany; 2 FFE Service GmbH, Feldkirchen, Germany; 3 Laboratory for Operation Control and Research, Zweckverband Landeswasserversorgung, Langenau, Germany; 4 Present Address: Bayerisches Landesamt für Umwelt, Augsburg, Germany

**Keywords:** Solid-phase extraction, Evaporative concentration, Hydrophilic interaction liquid chromatography, Non-target screening

## Abstract

For the analysis of low concentrations of micropollutants in environmental water samples, efficient sample enrichment and cleanup are necessary to reduce matrix effects and to reach low detection limits. For analytes of low and medium polarity, solid-phase extraction is used, but robust methods for the preconcentration of highly polar or ionizable analytes are scarce. In this work, field-step electrophoresis (FSE) was developed as an environmental sample cleanup technique for ionizable micropollutants and ionic transformation products. The FSE electrolyte system preconcentrated 15 acidic model analytes (pK_a_ from −2.2 to 9.1) present in aqueous samples in two fractions by factors of 5–10. Simultaneously, highly mobile matrix compounds were removed including inorganic ions such as sulfate and chloride. The fractions were either directly injected for downstream analysis by reversed-phase liquid chromatography (RPLC) or further processed by evaporative preconcentration with subsequent reconstitution in an organic solvent suitable for separation methods like hydrophilic interaction chromatography. The FSE/RPLC-MS method exhibited high quantitative precision with RSDs of 3–6%. The method was successfully applied to a spiked river water sample and its performance compared with common solid-phase extraction and evaporative concentration, demonstrating a high analyte coverage. FSE combined with non-target screening by RPLC-MS revealed a strong reduction in matrix load especially at low retention times. Seventeen compounds were identified in the FSE fractions sampled at the field step boundary by retention time, accurate mass, and mass fragments. Suspect screening by FSE/RPLC-MS was facilitated by FSE’s selectivity for anionic compounds.

**Supplementary Information:**

The online version contains supplementary material available at 10.1007/s00216-021-03856-w.

## Introduction

The pollution of surface waters by industrial waste, pharmaceuticals, and household chemicals requires extensive monitoring and thus sufficient analytical workflows to control governmental regulations and identify new risks. Since most micropollutants in the aquatic environment are present at trace levels (ng/l to μg/l range), even modern analytical instruments often do not reach the required limits of detection (LODs). Thus, different methods of sample enrichment were developed to increase the analyte concentration and/or reduce matrix interferences [1–4]. For screening purposes, the preconcentration method needs a low selectivity so that no analytes are discriminated by physicochemical characteristics such as polarity. Most work has been done using solid-phase extraction (SPE) [1, 3] or evaporative concentration (EC) [5] focusing on selected (classes of) analytes [6].

Recently, we compiled a list of 455 compounds previously detected in water and biota analysis from various research articles [7]. Among these compounds, 60% of the analytes were charged at pH 10, with 96% of them possessing a charge number ≤ −0.5 (values were simulated by Chemicalize provided by ChemAxon (11/02/2021)). Especially anionic compounds are of interest with regard to (drinking) water analysis as they are more mobile compared to cationic ones. In addition, transformation often introduces acidic functional groups, for example, by hydroxylation of aromatic subunits.

In this work, we transfer methods of free-flow electrophoresis (FFE) from protein analysis to environmental science, using the mode field-step electrophoresis (FSE). It was first introduced by Wagner and Kessler in 1983 [8] as a new method for preparative protein isolation. The basic principle is described by Weber et al. [9]. Briefly, FSE uses a flat separation chamber, filled with different electrolytes and the sample using parallel injection ports along the upper side. Contrary to common field zone electrophoresis, the separation buffer is not uniform across the separation chamber. Instead, the chamber is filled with two parallel bands of buffers strongly differing in their conductivity. The sample itself is introduced as a broad stream at the boundary into the low-conductivity section. The electric field is applied perpendicular to the buffer and sample flow in such a way that (in our case negatively) charged analytes migrate to the high-conductivity section. Once the ions reach the high-conductivity section, the migration velocity is reduced and the analytes become stacked at the boundary between the high- and low-conductivity buffers. The preconcentrated analytes can be sampled at the end of the separation chamber in different fractions.

The FSE principle offers several advantages for the analysis of environmental water samples: (i) the fractionation removes neutral and positively charged compounds. Fast migrating inorganic anions may be removed, too, as they are collected in different fractions when the focusing time and conductivity steps are optimized; (ii) high volumes of aqueous samples can be introduced and fractions collected continuously providing preconcentration from large sample volumes; (iii) in combination with volatile FSE media, enrichment factors (EFs) can be further improved by evaporation of fractions and reconstitution in smaller volumes; and (iv) depending on the subsequent separation method, an orthogonal separation mechanism (e.g., chromatography) can be applied.

In this study, the potential of FSE as cleanup and preconcentration step for environmental water samples was investigated and validated using reversed-phase liquid chromatography (RPLC-MS) and hydrophilic interaction liquid chromatography (HILIC–MS) as complementary chromatographic approaches [10]. A non-target screening for river water was conducted to evaluate potential interferences with the FSE media.

## Materials and methods

### Chemicals

1*H*-benzotriazole (BTA ≥ 98%), 2-methyl-4-chlorophenoxy acetic acid (MCPA ≥ 98%), 2-naphthalene sulfonic acid (2-NSA, ≥ 95%), 5-amino-2-naphthalene sulfonic acid (5-A-2-NSA, ≥ 95%), acesulfame (ACE, ≥ 99%), acetonitrile (MeCN, LC-MS grade), ethyl sulfate (ESU, ≥ 95%), formic acid (FA, 98%), hydrochlorothiazide (HCT, ≥ 99%), isopropanol (LC-MS grade), methanol (MeOH, LC-MS grade), saccharin (SAC, ≥ 98%), sulfamethoxazole (SULFA, ≥ 98%), sulfamic acid (SULAC, 99.3%), triethylamine (TEA, ≥ 99.5%), and water (LC-MS grade) were purchased from Sigma-Aldrich (Steinheim, Germany). Acetic acid (HAc, 100 %), ammonium acetate (NH_4_Ac, 98%), ammonium hydroxide (NH_4_OH, 25% aqueous solution, LC-MS grade), and dichloro acetic acid (DCAA, ≥ 98%) were obtained from Merck (Darmstadt, Germany). 4-(2-hydroxyethyl)-1-piperazine-ethane sulfonic acid (HEPES, 99.5%), 4-hydroxybenzoic acid (4-HBA, ≥ 98%), and umbelliferone (UMBE, 99%) were delivered by Fluka (Buchs, Switzerland). p-Toluene sulfonic acid (p-TSA, 90%) was purchased from Alfa Aesar (Haverhill, MA, USA), and hydrochloric acid (HCl, 32% aqueous solution) from Fisher Scientific (Waltham, MA, USA). Acetic acid (100 %) for FSE was delivered by Carl Roth (Karlsruhe, Germany). 4-Hydroxybenzoic acid-d4 (4-HBA d4), acesulfame-d4 potassium salt (ACE d4), dichloro acetic acid-d1 (DCAA d1), saccharin-^13^C6 (SAC ^13^C6), and p-toluene-d7-sulfonic acid H_2_O (p-TSA d7) were delivered by TRC (Toronto, Canada).

### Off-line FSE/LC-MS workflow

**Fig. 1:**
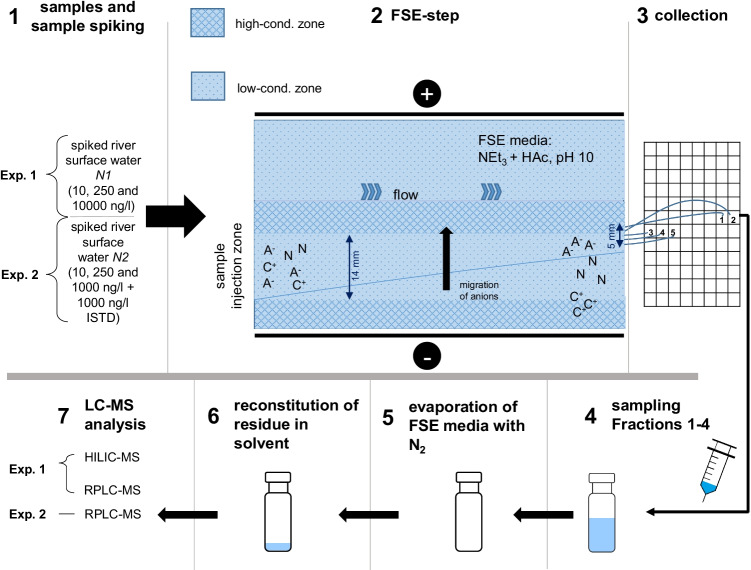
Workflow for the off-line FSE/LC-MS experiments. High-conductivity zone: 250 mM triethylamine (TEA) + HAc, pH 10.3, low-conductivity zone: 15 mM TEA + HAc, pH 10.3. A^−^: anionic compounds, C^+^: cationic compounds, N: neutral compounds

The basic workflow of FSE with subsequent analysis by LC-MS is presented in Fig. 1. For FSE separation, aqueous standards or river water spiked with model analytes and isotope-labeled standards (Step **1**, Fig. 1, see “Preparation of solutions” and “Spiking for the determination of LODs and matrix effects”) were continuously injected for 2.2–2.3 min (Step **2**, see “FSE experiments”) into the low-conductivity buffer as a broad zone. Five fractions (**F**_**1**_–**F**_**5**_, corresponding to Fractions 55–59 on the 96-dwell plate in the FSE setup, see Fig. 1) were collected, which included the preconcentration zone at the stacking boundary as well as neighboring fractions. Due to stacking, the original sample zone of anionic analytes was narrowed. The fractions were then prepared for subsequent analysis according to “Treatment of FSE fractions” (Steps **3**–**6**) or for preconcentration by evaporation and reconstitution. The final analysis (Step **7**) was conducted using HILIC-MS or RPLC-MS for the first set of experiments (**Exp. 1**). In a second set of experiments (**Exp. 2**), analytes were spiked to river water at different concentrations and isotope-labeled standards were added. Fractions from **Exp. 2** were analyzed using RPLC-MS. Steps **2**–**5** were identical for all experiments.

### Samples and sample preparation

#### Collection of river water

Two river water samples *N1* and *N2* were collected from the river Neckar in Tübingen, a few hundred meters downstream of a wastewater treatment plant (WWTP, 110,000 population equivalents) in February and September 2020. Samples were collected in polypropylene vessels, filtered with a CHROMAFIL Xtra PTFE-45/25 filter (Macherey-Nagel, Düren, Germany), and stored in a borosilicate vessel at −20 °C before use.

#### Preparation of solutions

Methanolic stock solutions with a concentration of 20 mg/l containing all analytes (see Table 1) were prepared using 1 g/l methanolic stock solutions of each analyte. Isotope-labeled standards (ISTD, deuterated and/or ^13^C-labeled) were prepared and stored in the same way. Water (for RPLC) or MeCN (for direct HILIC analysis) and FSE matrices were spiked with the analyte and ISTD mixtures, to reach a constant ratio of analyte mix:sample solution of 1:99 to keep the methanol content low and constant. Stock and working solutions were stored at −20 °C before use.

**Table 1:**
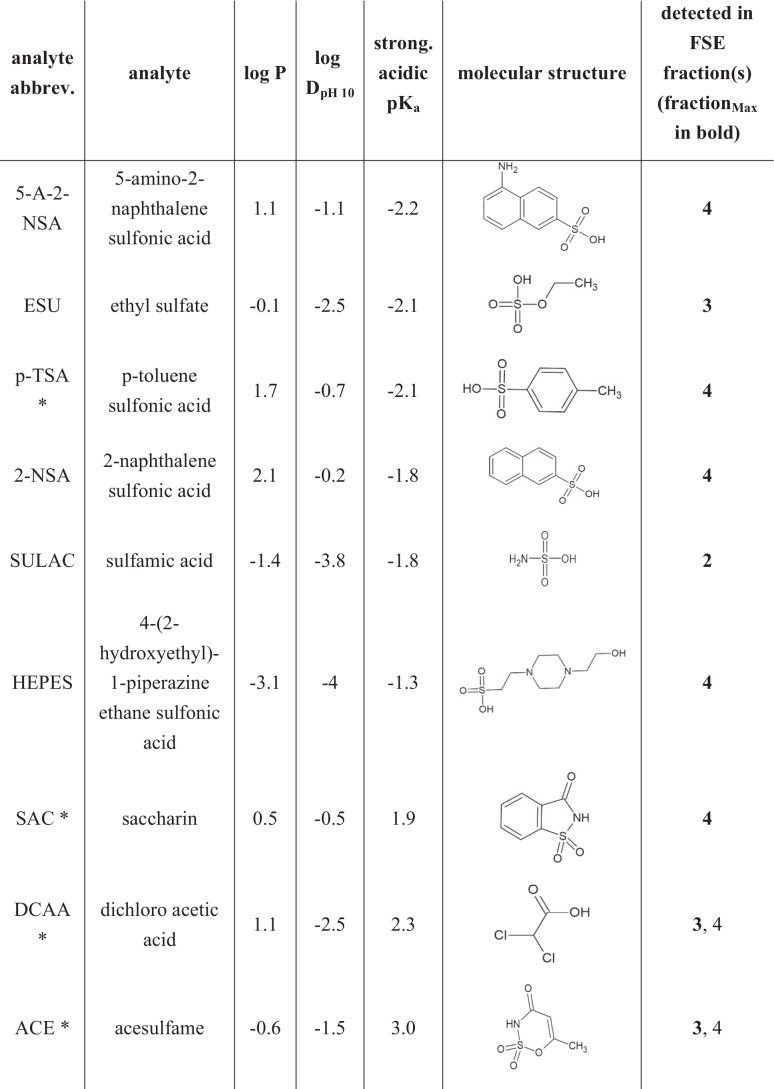

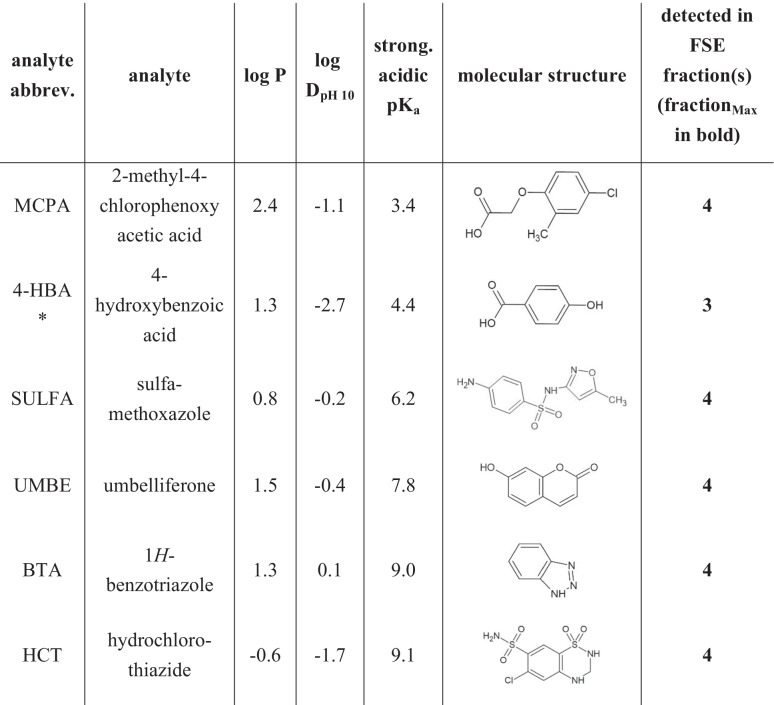
Model analytes and their physicochemical properties sorted according to lowest (acidic) *pK*_*a*._

*Isotope-labeled standard available, see “*Chemicals*.” pK_a_, log *P*, and log *D*_pH10_ values were simulated by Chemicalize provided by ChemAxon (11/02/2021)

#### Treatment of FSE fractions

After the FSE experiments, selected fractions were filtered via CHROMAFIL Xtra PTFE-45/25 filters (Macherey-Nagel, Düren, Germany), pooled if necessary, and evaporated to dryness under a gentle stream of nitrogen. The concentrated residue was reconstituted in the same volume of a suitable solvent, if not stated otherwise. The type of solvent was adapted to the subsequent analysis technique (RPLC-MS: H_2_O, HILIC-MS: MeCN/H_2_O (95:5, v/v)).

#### Spiking for the determination of LODs and matrix effects

Blank FSE fractions (FSE experiment using LC-MS grade H_2_O as sample) were spiked to estimate the method LODs and determine matrix effects by FSE buffer components. In preliminary experiments (data not shown), we were able to show that Fractions **F**_**3**_ and **F**_**4**_ hold the major share of model analytes and only the neighboring fractions also contained analytes (neighboring fractions **F**_**1**_, **F**_**2**_, and **F**_**5**_), albeit mostly at low concentrations. These five fractions covered a width of 5 mm in the FSE setup compared to the sample inlet of 14 mm. The fractions were evaporated to dryness under a gentle stream of nitrogen after filtration. For some experiments, fractions were pooled (for example, Fractions **F**_**3**_–**F**_**5**_, called **F**_**3–5**_). Spiking concentrations had to be adapted to the downstream analysis: HILIC-MS: 10, 100, 500, 1000, and 2500 ng/l; RPLC-MS: 100, 1000, 5000, and 10,000 ng/l. Matrix effects were determined by comparing peak areas of the analytes spiked to FSE fractions vs. direct injection of an aqueous standard. Concentrations of the aqueous standard were 500, 1000, and 10,000 ng/l for HILIC-MS and RPLC-MS.

For the comparison of FSE preconcentration capabilities with SPE and EC (see “Comparison of FSE/RPLC-MS with common SPE and EC”), 500 μl of the pooled Fraction **F**_**3–4**_ of **Exp. 2** were evaporated to dryness as described. Reconstitution followed in 50 μl LC-MS grade H_2_O (thus volume reduction by a factor of 10) and samples were analyzed using RPLC-MS.

#### FSE experiments

The FFE instrument consists of a horizontal separation chamber with the dimensions 500 mm × 100 mm. A spacer of 0.2 mm was used to provide a thin film of an aqueous separation medium formed between the top and bottom plate. Two electrodes are placed at the sides of the cell to apply a high voltage perpendicular to the laminar flow introduced at the top by peristaltic pumps. Convective mixing is low at these channel dimensions. Thermoconvection was reduced using horizontal separation chambers with cooling from the bottom. A remaining temperature gradient then aids in stabilizing the streaming layers.

FSE separations were conducted at 10 °C using the following conditions and media: A flow rate of approx. 330 ml/h was used in combination with a voltage of 600 V, which resulted in a current of approx. 90 mA. The FSE chamber was filled with a high- and low-conductivity buffer made of 250 or 15 mM triethylamine, both titrated to pH 10.3 using acetic acid. The positioning of the solutions in the chamber is indicated in Fig. 1. Samples were perfused into the low-conductivity buffer zone at 12.7 ml/h. Residence time in the separation chamber was 2.2–2.3 min. Fractions were collected (collection rate of 3.4 ml/h) in polypropylene microtiter plates, numbered 1 (anode) to 96 (cathode). The ratio of sample rate (12.7 ml/h) and fraction collection rate (3.4 ml/h) led to a volume enrichment factor (VEF) of 3.7 by FSE. This value corresponds to the enrichment factor theoretically achieved if an analyte is completely focused in only one FSE fraction. Fractions labeled 1 and 2 were collected in the high-conductivity zone, Fractions 3–5 were collected from the low-conductivity zone (see Fig. 1), both directly near the boundary between these zones, where enrichment takes place.

The FSE experiments were conducted in two experimental blocks using sample *N1* in **Exp. 1** and sample *N2* in **Exp. 2** (see Fig. 1). In **Exp. 1**, we used the blank sample *N1* (*N1-blank*), and *N1* spiked with analytes at concentrations of 10, 250, and 10,000 ng/l (labeled *N1-10, N1-250, N1-10,000 ng/l*) to cover a wide concentration range and enable the analysis using HILIC. **Exp. 2** was conducted for subsequent target and non-target screening by RPLC-MS: the raw sample *N1* was injected for FSE directly *(N1)*, or spiked at concentrations of 10, 250, and 1000 ng/l (*N1-10, N1-250, N1-10,000 ng/l*), for downstream RPLC-MS. Additionally, an aqueous sample spiked at 250 ng/l (*H*_*2*_*O-250 ng/l*) was subjected to FSE and used as a reference. In **Exp. 2**, ISTD were spiked at a concentration of 1000 ng/l before FSE experiments in order to determine precision and to investigate if the preconcentration depends on the concentration in the sample. A system blank was obtained injecting LC-MS grade H_2_O for FSE fractionation (*H*_*2*_*O-blank*).

#### SPE procedure

To maximize analyte retention on the SPE material, 5 ml of sample *N2* (spiked with concentrations of 10, 250, and 1000 ng/l of analyte mix and 1000 ng/l ISTD mix as additional analytes in all samples) was acidified to pH 1 with HCl. Prior to loading, the cartridge (30 mg Oasis HLB, Waters, Eschborn, Germany) was washed three times with 1 ml MeOH (LC-MS grade) and conditioned three times with 1 ml water (LC-MS grade). The highest extraction efficiencies (results not shown) for model analytes (see Table 1) were reached using an elution medium of 5% aqueous NH_3_ solution (25%) in MeOH without washing steps after loading. The eluate was evaporated to dryness under a gentle stream of nitrogen, and the concentrated residue was redissolved in 0.5 ml H_2_O (relative enrichment factor of 10). Finally, the sample was injected for RPLC-MS analysis.

#### EC procedure

One milliliter of river sample *N2* (spiked with concentrations of 10, 250, and 1000 ng/l of analyte mix and 1000 ng/l ISTD mix as additional analytes in all samples) was evaporated to dryness under a stream of nitrogen and redissolved in 0.1 ml H_2_O (relative enrichment factor of 10). All samples were analyzed by RPLC-MS.

### Separation techniques

#### LC-MS analysis

The RPLC-MS method used for non-target screening (referred to as *RPLC-NTS*, “Applicability of FSE as sample cleanup step for non-target screening of acidic compounds”), was described previously [11]. Briefly, the stationary phase used was the same as described in “RPLC-MS”. In contrast to previous work, an x500R-System (Q-TOF, Sciex Applied Biosystems, MA, USA) was used; MS parameters are summarized elsewhere [12]. Ninety-five microliters of diluted sample were mixed with 5 μl of a solution containing 16 isotope-labeled control standards for internal control measures before injection [11].

For all other RPLC-MS and HILIC-MS analyses, a 1260 Infinity LC system coupled to a 6550 iFunnel Q-TOF LC/MS system (Agilent Technologies, Waldbronn, Germany) was used. A jet-stream electrospray ionization (ESI) source was operated with a nebulizer pressure of 35 psi, a drying gas temperature of 160 °C, a flow rate of 16 l/min, and a fragmentor voltage of 360 V. In the negative ionization mode, the capillary voltage was set to 4000 V, the skimmer voltage to 65 V, and the nozzle voltage to 500 V. The mass range was 40–1000 *m*/*z* with a data acquisition rate of 1 spectrum/s. The sheath gas temperature was set to 325 °C with a flow rate of 11 l/min. For internal calibration, solutions of purine and HP0921 (Agilent Technologies, Waldbronn, Germany, *m*/*z* = 121.0508, 922.0097) in MeOH/water (95/5) were used and sprayed via a reference nebulizer.

##### RPLC-MS

Aliquots of 10 µl of the processed samples were injected onto a Zorbax Eclipse Plus C18 column (2.1 × 150 mm, 3.5 μm, narrow bore, Agilent Technologies, Waldbronn, Germany) for the analysis of compounds of medium polarity. Additionally, a guard column (2.1 × 15 mm, 5 μm, narrow bore, Agilent Technologies, Waldbronn, Germany) was used. For separation, a gradient elution at a flow rate of 0.3 ml/min using (A) water and (B) MeOH, both containing 0.1% FA (v/v), was chosen. After 1 min, the initial content of 95% water was decreased to 5% water over 7 min. This mobile phase was kept for another 7 min. Then, the water content was increased to 95% over 5 min.

##### HILIC-MS

Aliquots of 5 µl  of the processed samples were injected onto a SeQuant ZIC-HILIC column (2.1 × 150 mm polyether ether ketone (PEEK) coated, 3.5 μm, 100 Å, Merck, Darmstadt, Germany) for the analysis of polar compounds. In addition, a guard column (2.1 × 20 mm PEEK coated, Merck, Darmstadt, Germany) was installed in front of the column with a coupler. For separation, a gradient elution at a flow rate of 0.3 ml/min using (A) aqueous 20 mM NH_4_HCO_3_ and (B) MeCN, both containing 0.01% FA (v/v), was chosen. The initial content of 90% MeCN was decreased to 40% water over 15 min. This mobile phase was kept for 1 min and the MeCN content was increased to 90% over 0.5 min. To ensure full re-equilibration, this composition was kept for another 8 min before injecting the next sample, leading to a total analysis time of 24.5 min. The re-equilibration step used an increased flow rate of 0.5 ml/min between 16 and 22 min.

### Data processing and method performance tests

For method development and performance testing, ion chromatograms (EICs) were extracted and evaluated from mass profile data with a mass accuracy of 0.01 *m*/*z* using Mass Hunter Qualitative Software (Agilent, V10.0). S/N ratios used in “Comparison of FSE/RPLC-MS with common SPE and EC” were also calculated using Qualitative Software. MassHunter Quantitative Software (V10.1) was used to create calibration curves. In order to estimate the extent of matrix effects, the peak areas of spiked analytes were compared in blank FSE fractions to the peak area of an aqueous standard (500 or 1000 ng/l for HILIC and RPLC). Enrichment factors (EFs) were calculated by the ratio of the peak areas (for non-target screening peak heights) determined in FSE fractions vs. in spiked native samples. EF values thus comprised effects from enrichment, possible losses, and matrix effects. Fractions with the highest analyte concentration are referred to as fraction_MAX_ for each analyte.

Data were evaluated with Origin 2020 (OriginLab Corporation, Northampton, USA) and Microsoft Excel 2019 (Microsoft Corporation, Redmond, WA, USA). Statistical evaluation was conducted using IBM SPSS Statistics 26 (IBM, Armonk, NY, USA).

## Results and discussion

### Study design

As a first step (**Exp. 1**, see Fig. 1), the compatibility of FSE fractionation with downstream RPLC-MS and HILIC-MS was investigated using the river water sample *N1* spiked with model analytes (10, 250, and 10,000 ng/l). RPLC-MS performed best and was used for subsequent experiments. The performance of FSE sample pretreatment was evaluated in a second set of experiments (**Exp. 2**, see Fig. 1) by comparison with common SPE and EC sample preparation using the spiked river water sample *N2*. With the optimized method, FSE fractions of river water were analyzed by non-targeted RPLC-MS.

### Model analyte system

Twenty model analytes were chosen due to the broad range of physicochemical characteristics covered: the analytes differed in their functional groups (e.g., sulfonamides, sulfonic acids (halogenated), carboxylic acids, amines), and thus polarity (−3.1 ≤ log *P* ≤ 2.4) and acidity (−2.2 ≤ pK_a_ ≤ 9.1). For polarity, we considered log *D* at pH 10 according to the pH in the FSE experiments. The log *D*_pH 10_ range was from −4.0 to 0.1. To judge the FSE preconcentration effects and precision in river water samples, we spiked five isotope-labeled standards ISTD at a concentration of 1000 ng/l. The ISTD were used to (1) account for endogenous compounds in the sample, (2) to judge the concentration dependence of FSE, and (3) to have additional analytes.

### Compatibility and orthogonality of FSE with common subsequent separation techniques

The compatibility with RPLC-MS and HILIC-MS was investigated. We also tested capillary electrophoresis coupled to mass spectrometry but limits of detection were not sufficient. A direct injection of FSE fractions was possible for all methods, but triethylamine from the FSE electrolyte caused a high background. It was partially removed by evaporating to dryness and reconstituting the fractions as described in Fig. 1 (Steps **5** and **6**). After evaporation and reconstitution for RPLC-MS and HILIC-MS, matrix effects (see “Data processing and method performance tests”) were about 100% on average demonstrating a negligible matrix (see supporting information). For the model analytes chosen, method LODs were lowest for separations using HILIC (0.10–0.25 μg/l) and slightly elevated for RPLC (0.6–0.8 μg/l). Surely, increasing the injection volume in RPLC-MS would further improve LODs. Overall, FSE proved to be well compatible with HILIC and RPLC separation.

**Fig. 2:**
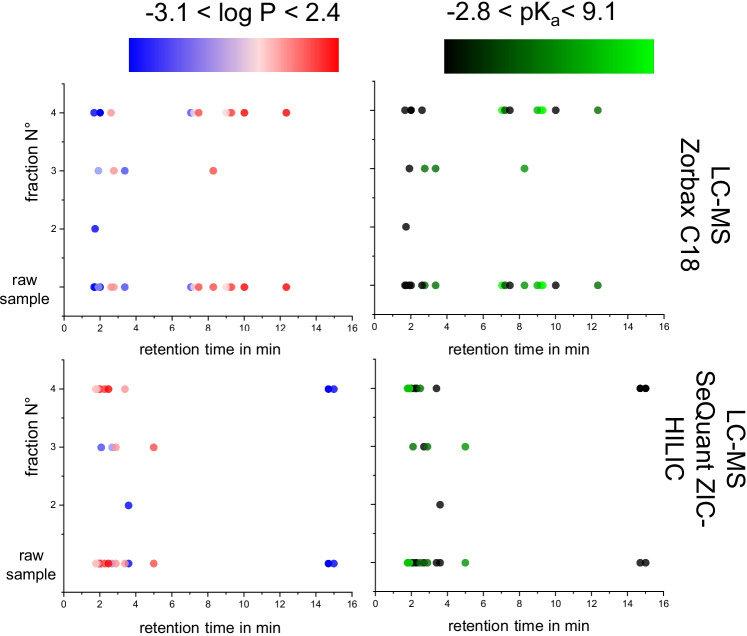
Differences in selectivity of FSE fractionation (Fractions **F**_**2**_–**F**_**4**_) vs. RPLC and HILIC, representing analytes by their retention times. Circles represent analytes detected in their specific fraction_MAX_ (FSE separation) or in the raw sample (for comparison) plotted with their retention time. The color code refers to polarity or pK_a_ values. With HILIC-MS, BTA and DCAA were not detected. As the pH of the separation media was different in FSE and LC, we chose log *P* for this comparison

We investigated the selectivity of FSE with regard to analyte polarity or pK_a_ values and assessed the orthogonality of preparative FSE and downstream analytical separation. In Fig. 2, we compare which analytes are present in the reference sample vs. fractions, representing the analytes via their retention times (RT). Their log *P* and pK_a_ values are indicated by a color code. As expected, selectivity is mainly dominated by polarity for chromatographic separations. No correlation between FSE fractionation and elution order of the analytes was present. This is best visible by the broad distribution of retention times in Fraction **F**_**4**_ covering analytes with a broad range of polarities and pK_a_ values. However, Fractions **F**_**2**_ and **F**_**3**_ contain most of the early eluting analytes.

Even though analyses using HILIC-MS offered the lowest method LODs, RPLC was used for further performance testing, as all 15 model analytes were the uniformly distributed over the separation window (see Fig. 2) and sufficient sensitivity was reached. With HILIC-MS, only 13 of the 15 analytes were detected as method LODs were not met for DCAA and BTA.

### FSE fractionation and reproducibility

**Fig. 3:**
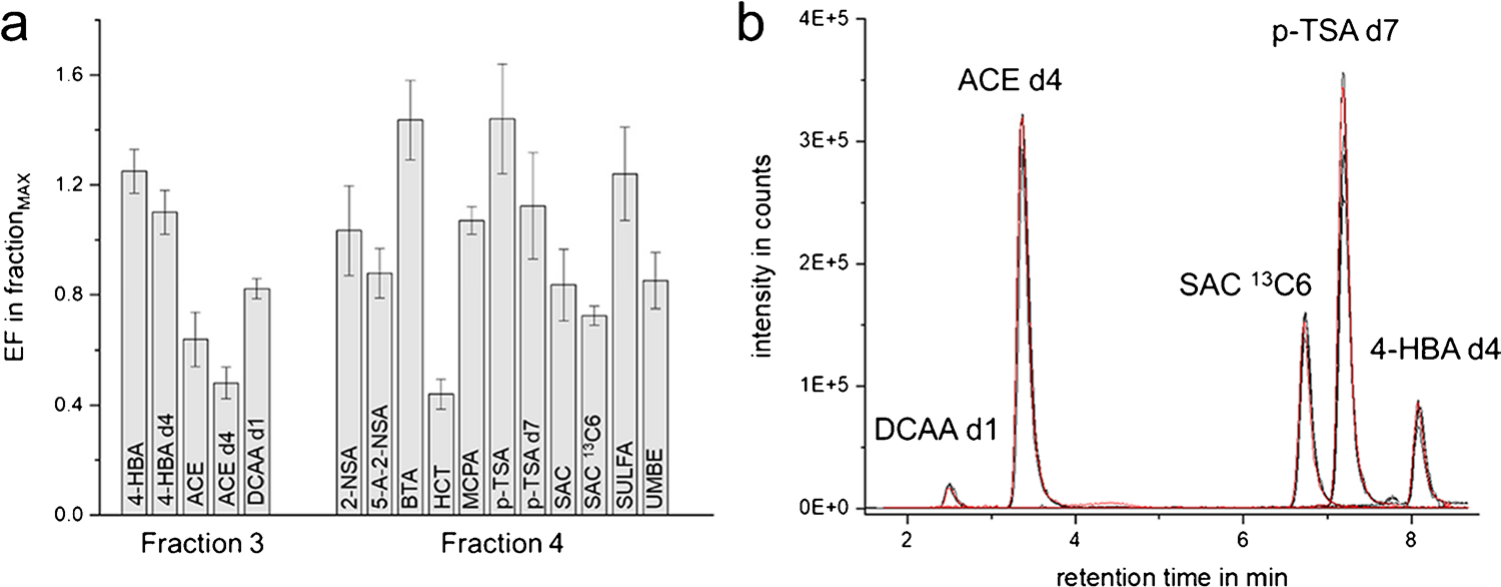
**a** Average relative enrichment factors of analytes in the fraction_MAX_ relative to the peak area of the original spiked water samples (directly injected) for 16 of the 20 analytes, calculated from RPLC-MS analysis. For model analytes, EFs of the FSE experiments N1-250/10,000 ng/l and N2-250/1000 ng/l were considered (*n* = 4). For the ISTD, results from **Exp. 2** (N2, N2-10, N2-250, N2-1000 ng/l, and H_2_O-250 ng/l, thus *n* = 5) are plotted. For ESU, DCAA, and HEPES, LODs were not reached, for SULAC signal overloading was observed in the original sample. **b** Comparison of the chromatograms of spiked FSE fraction_MAX_ samples of river water N2 and an aqueous standard (Fraction **F**_**3**_: DCAA d1, ACE d4, and 4-HBA d4; Fraction **F**_**4**_: SAC ^13^C6 and p-TSA d7). Fractions of all five samples (N2, N2-1, N2-250, and N2-1000 ng/l, H_2_O-250 ng/l) were prepared as described in “Treatment of FSE fractions” and analyzed by RPLC-MS (see “RPLC-MS”)

Analytes were preconcentrated at the boundary between high- and low-conductivity buffer zones leading to very few fractions with elevated concentrations of the analytes (see Fig. 1). Thirteen of the 15 analytes and internal standards were determined in Fraction **F**_**4**_ by LC-MS, except SULAC and 4-HBA which were detected in Fractions **F**_**2**_ and **F**_**3**_, respectively. DCAA and ACE were detected in Fractions **F**_**3**_ and **F**_**4**_ (ratios of peak areas **F**_**3**_/**F**_**4**_ DCAA 70:30 and ACE 60:40) (see Table 1). For all other analytes, F_4_ was fraction_MAX_. The average relative EFs in Fractions **F**_**3**_ and **F**_**4**_ were 0.86 and 1.01. Fig. 3a exemplarily shows the average EFs in the Fractions **F**_**3**_ and **F**_**4**_ of the 16 model analytes which were detected at all three concentration levels.

We calculated the EFs at the concentration level of 10,000 ng/l for fraction_MAX_ vs. the spiked raw water sample. EFs were between 0.4 and 1.4, demonstrating that both enrichment and matrix effects and possible analyte losses have to be considered: compared to the theoretical *VEF* of 3.7 of the FSE fractionation (see “Data processing and method performance tests”), this means that 11–50% of an analyte were present in fraction_MAX_. The sum of EFs over all fractions was in the range of 0.6 to 2.3 equaling 16-70% of an analyte.

The reproducibility of the whole method was determined using water and the river water samples N2 spiked with 1000 ng/l in **Exp. 2**. First, the fractions were analyzed using RPLC-MS. Mostly, Fractions **F**_**3**_ or **F**_**4**_ were fraction_MAX_ (see Table 1). The high reproducibility is well documented by Fig. 3b with an overlay of five EICs for each analyte in fraction_max_, reaching RSD values between 3 and 6% over the entire analytical process.

The precision was independent of the analyte concentration: EFs of analytes spiked at different concentrations and their isotope-labeled counterparts (fixed concentration) were not significantly different. The error bars in Fig. 3 reveal very good average RSDs of 9 and 12% for analytes’ peak areas in Fractions **F**_**3**_ and **F**_**4**_ for two separate analyses using both *N1* and *N2* within a time interval of over half a year.

Clearly, a large analyte coverage was reached spreading over a polarity range of −3.1 ≤ log *P* ≤ 2.4 and acidity range of −2.2 ≤ pK_a_ ≤ 9.1. The only major selectivity criterion was the negative charge of the analytes. As the FSE experiment was performed at pH 10, even less acidic compounds, e.g., HCT (pK_a_ = 9.1) are addressed.

### Comparison of FSE/RPLC-MS with common SPE and EC

We compared the sample preparation by FSE with evaporative concentration and solid-phase extraction as common alternatives. For SPE, Oasis HLB cartridges were chosen as an accepted standard in LC-MS approaches for a wide polarity range of analytes [6]. A preconcentration factor of 10 was chosen for all techniques by volume reduction to better compare results with regard to matrix effects. The detailed protocols are given in “Off-line FSE/LC-MS workflow.”

**Fig. 4:**
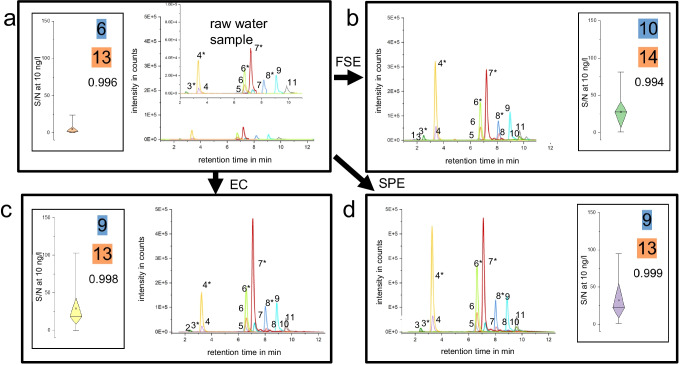
RPLC-MS chromatograms of **a** the raw sample N2-10 ng/l, **b** of the pooled FSE Fraction **F**_**3–4**_ (see “Spiking for the determination of LODs and matrix effects” and “FSE experiments”), **c** of the SPE extract (see “SPE procedure”), and **d** of the EC reconstituted solution (see “EC procedure”). The inserts show (i) blue: number of analytes at *c* = 10 ng/l, (ii) orange at *c* = 1000 ng/l, (iii) the mean *R*^2^ values of the calibration curve for all analytes, and (iv) box-whisker plots for average S/N at *c* = 10 ng/l. Signals: (1) HEPES, (2) ESU, (3) DCAA, (4) ACE, (5) HCT, (6) SAC, (7) p-TSA, (8) 4-HBA, (9) BTA, (10) UMBE, and (11) 2-NSA and * their isotope-labeled standards at *c* = 1000 ng/l in the raw sample N2. SULAC was excluded due to strong quenching effects (results not shown) for all three sample preparation methods

Fig. 4 shows the EICs of analytes of the spiked river water sample N2-10 ng/l before and after sample preparation by FSE, SPE, and EC (Fig. 4a–d).

#### Selectivity and sensitivity

We here look at relative values between samples with vs. without sample preparation as some analytes were endogenously present in the river water, e.g., 2-NSA, ACE, BTA, HCT, p-TSA, and SAC. All sample preparation methods provided an increase in sensitivity with increased S/N ratios and a higher number of analytes detected already at spiking concentrations of 10 ng/l (see blue numbers in Fig. 4). The increase of S/N values proved significant with a factor of approx. 6 for all three sample preparation techniques (paired *t*-test, *α* = 0.05) compared to the untreated sample. FSE media had only minor effects on LODs, which was shown by spiking analytes to blank FSE media (data not shown).

Looking at specific analytes, signals of 4-HBA and UMBE increased after all three sample preparation steps investigated here and became detectable even at the lowest spiking concentration of 10 ng/l (before preconcentration only at 250 ng/l), so we can presume that the enrichment was close to the theoretical factor of 10 for SPE and EC. Results were similar for all sample preparation techniques for SULFA, MCPA, and 5-A-2-NSA (at spiked concentrations ≥ 250 ng/l). Matrix effects seemed to impair SULAC analysis. ESU, HEPES, and DCAA were enriched differently: ESU became detectable after preconcentration only at elevated concentrations (≥ 250 ng/l) using EC and FSE. Using SPE, a decrease in peak area compared to the original sample was observed caused by the poor retention of the ionic compound on the SPE sorbent. DCAA was enriched by all three sample preparation steps. However, strong quenching effects were present after EC, probably caused by co-enrichment of matrix compounds inherent to this technique. Preconcentration of DCAA was effective by SPE and FSE enabling its analysis at concentrations below 10 ng/l. The analyte HEPES could only be quantified after FSE but not after SPE or EC, eventually due to ionization quenching by matrix components (see “Compatibility and orthogonality of FSE with common subsequent separation techniques”), or a strong positive bias in the enrichment process either in FSE or negative bias in SPE and EC. For a broader view on the selectivity and efficiency of FSE, further investigations with more model analytes will aid to fully understand all parameters influencing the FSE fractionation.

#### Comparison of preconcentration techniques

Our results show an improvement in sensitivity by all three sample preparation techniques. The newly established FSE showed some bias regarding analytes’ charge state, though less pronounced as the bias of SPE regarding analytes’ polarity. In general, analyte losses by SPE and FSE are expected to be higher compared to EC, as the latter will only lose volatile components or analytes which are difficult to redissolve after evaporation. FSE sample treatment offers several advantages: the efficient salt removal increases the loadability of samples compared to EC with its increasing salt loads and strong matrix effects. Due to the fractionation by FSE, large concentration differences in the sample are not critical and overloading, as sometimes present in SPE, is prevented. The abandonment of organic solvents is favorable, and FSE shows high potential for automation. FSE and RPLC-MS are orthogonal in their separation process, and matrix effects were clearly lowered (see “Compatibility and orthogonality of FSE with common subsequent separation techniques”).

### Applicability of FSE as sample cleanup step for non-target screening of acidic compounds

Fractions **F**_**1**_ and **F**_**4**_ and the spiked river sample from **Exp. 2** were analyzed with an established *RPLC-NTS* method (see “LC-MS analysis”) optimized for the non-target screening of micropollutants in environmental waters [11]. We investigated the extent of matrix removal and the use of charge as an additional identification criterion to verify suspects and minimize false-positive results.

#### Removal of matrix components

**Fig. 5:**
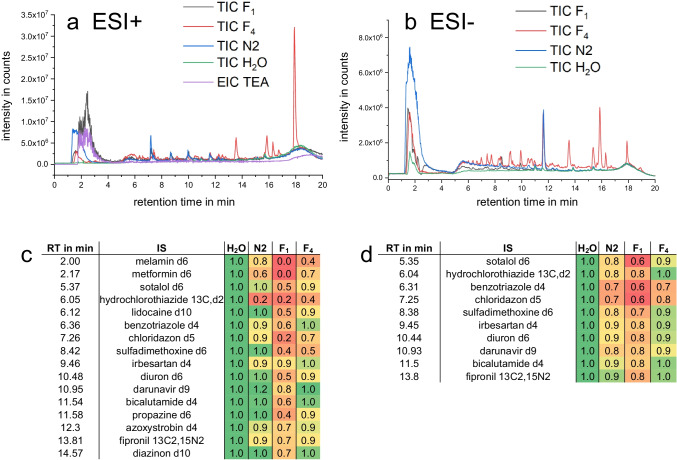
**a** and **b** TICs of a blank sample (LC-MS grade H_2_O, spiked with control standards, green mass chromatograms), of the river water samples N2-250 ng/l and N2-1000 ng/l (blue lines) spiked with the model analytes as well as the TICs for FSE fractions (**F**_**1**_, of sample N2-250 (ESI+) with N2-1000 ng/l (ESI−, black lines) and **F**_**4**_ of N2 (red lines)) in both positive (**a**, **c**) and negative (**b**, **d**) ESI polarity. In positive ESI polarity, the EIC of triethylamine (TEA) is plotted in purple. **c** and **d** show a heat map with peak heights of the spiked IS normalized to 1 for different matrices (H_2_O, N2, **F**_**1**_, and **F**_**4**_). The RPLC-NTS method described in “LC-MS analysis” was used. All samples were diluted one-fold

Fig. 5a and b show the total ion chromatograms (TICs) of the river water sample *N2* and fractions thereof in positive and negative ESI mode. A significant decrease in the intensity of the matrix components eluting close to or in the void volume was visible by the reduction of (inorganic) salts and very polar neutrals using FSE prefractionation.

Only compounds in the sample stream directed to Fractions **F**_**3**_ and **F**_**4**_ were recovered by FSE for subsequent RPLC-MS analysis. Neutral compounds remained in the sample zone but were not preconcentrated at the stacking boundary. Cationic compounds migrated away from the stacking boundary and anions of high electrophoretic mobility, e.g., the inorganic anions chloride, phosphate, and sulfate, often present at high concentrations in environmental samples crossed the stacking boundary and were not present in the fractions of interest at elevated concentration. A further optimization is possible changing the relative sample flow rate and the migration path length in FSE: a shorter migration path length speeds the separation process and aids in sample stacking. With the current setup using about half the separation chamber for separation, the sample amount injected could be doubled. Obviously, sample cleanup using FSE was successful in reducing the number of matrix components eluting in or close to the void volume, as intensities in Fraction **F**_**4**_ decreased significantly compared to the raw sample. The only exception was the TIC of Fraction **F**_**1**_ analyzed in the positive ionization mode. Here, the maximum of the TIC signal at the beginning of the chromatogram was at higher retention times. Triethylamine, the cation in the FSE electrolytes, was identified as the major contributor to this signal as can be seen by the EIC of TEA at *m*/*z* 102.128 in Fig. 5a. A high amount in **F**_**1**_ is expected, which is sampled in the high-conductivity zone with its 17-fold higher concentration compared to the low-conductivity zone which was sampled in **F**_**4**_.

Fig. 5c and d depict two heat maps coding the signal suppression of the control standards spiked to every sample just before *RPLC-NTS* analysis in both ESI modes (reference was pure water spiked with the control standards). Matrix interferences were acceptable in positive ESI mode and were slightly reduced upon FSE sample preparation in negative ESI mode compared to the natural sample. The heat maps reveal different matrix effects for the different analytes in the river water sample vs. the two Fractions **F**_**1**_ and **F**_**4**_ using positive ESI polarity (Fig. 5c). Analysis using negative ESI mode (Fig. 5d) revealed stronger matrix effects. Overall, matrix effects by the FSE medium were small compared to matrix effects from the sample matrix.

#### Non-target screening

**Fig. 6:**
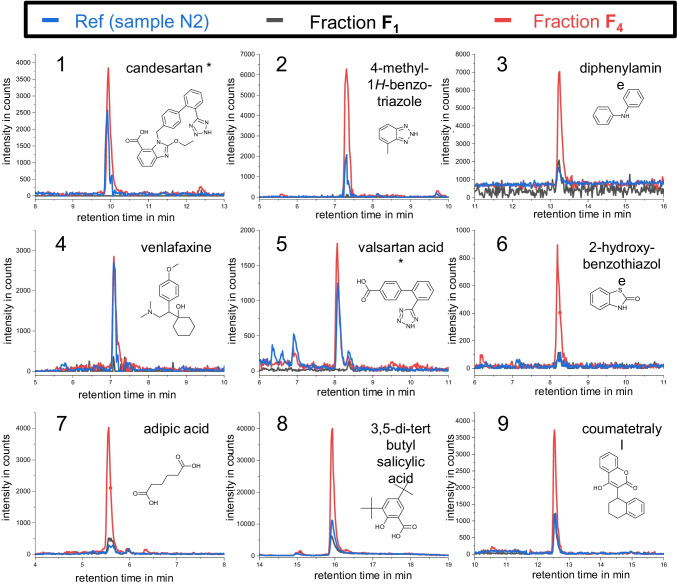
EICs of selected suspects in the sample N2 (blue lines) and the Fractions **F**_**1**_ (black lines) and **F**_**4**_ (red lines) (confirmed by their MS/MS spectra including their molecular structures) listed in Table 2 using RPLC-NTS. For analytes marked with an asterisk, MS/MS spectra are depicted exemplarily in Fig. 7. For details on RPLC-NTS method parameters, the reader is referred to [11]

**Fig. 7:**
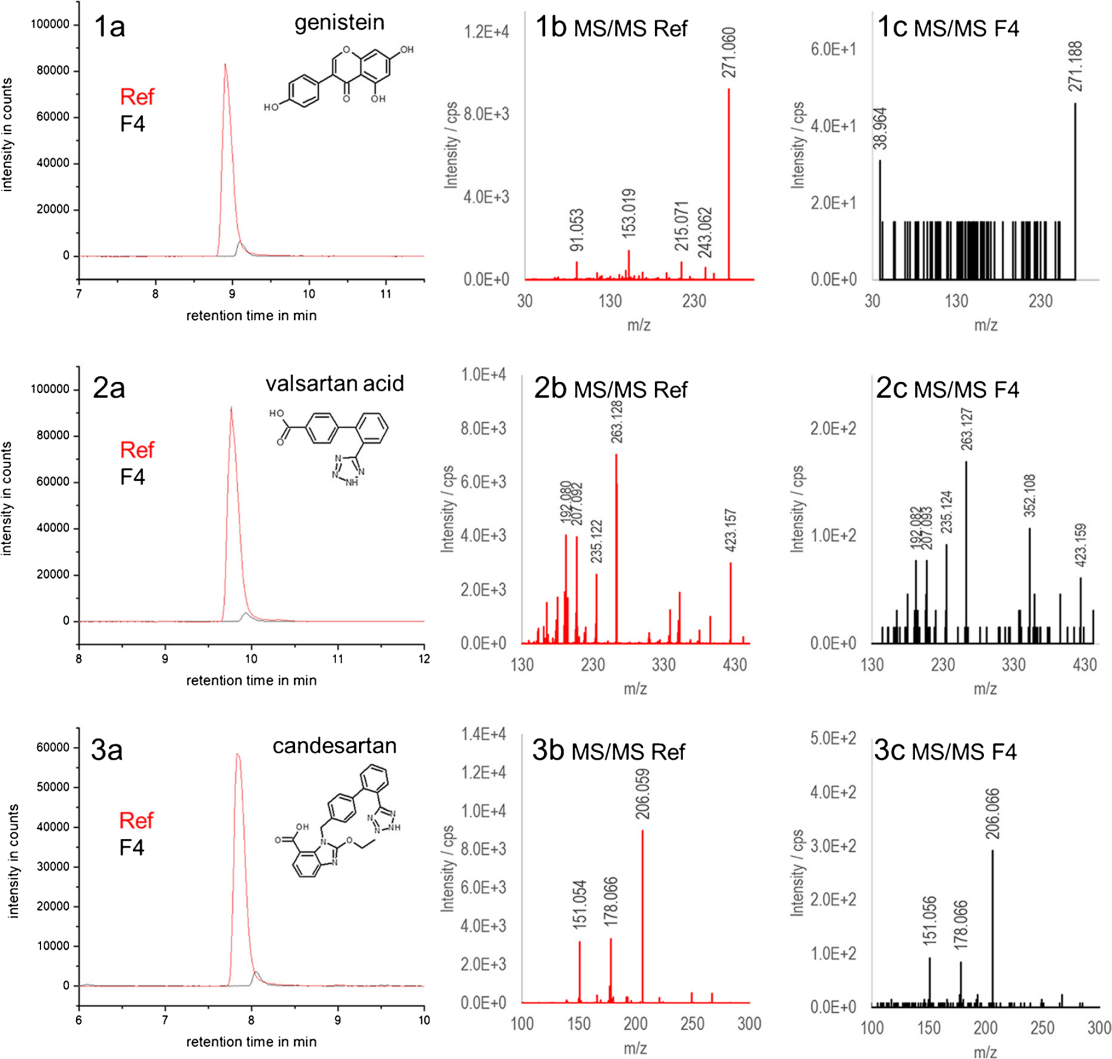
EICs of the suspects genistein (1, not verified by MS/MS), valsartan acid (2, verified by MS/MS), and candesartan (3, verified by MS/MS) also listed in Table 2. EICs of reference standard (pink) and suspect in Fraction **F**_**4**_ are shown in 1a, 2a, and 3a. MS/MS spectra of the suspects are given in Panels 1b, 2b, and 3b from the reference standard (5 μg/l) and in Panels 1c, 2c, and 3c from Fraction **F**_**4**_. Comparison of MS/MS spectra to reference spectra falsified suspect genistein and verified suspects valsartan acid and candesartan. For details on RPLC-NTS method parameters, the reader is referred to [11, 12]

Already in the TIC traces of Fraction **F**_**4**_, an increase in intensity and number of distinct signals was observed for both ESI polarities (see Fig. 5a and b). A closer evaluation of the data using libraries revealed 26 suspects based on retention time and exact mass. The suspects are listed in Table 2 and some EICs are shown in Fig. 6. Suspect analytes were only detected in Fraction **F**_**4**_ and were enriched by factors between 0.9 and 15.2 (see ratio **F**_**4**_/sample in Table 2). The suspects were investigated in more detail considering the criteria of FSE of anionic charge and a theoretical VEF of 3.7. For some analytes, EFs > 3.7 were observed which cannot result solely from FSE preconcentration (e.g., adipic acid (EF = 15.2), N°7 in Fig. 6). Matrix effects and low precision at concentrations close to the LOD may account for too high EFs. However, some suspects, e.g., levetiracetam and genistein, exhibited EFs of over 100, clearly indicating false-positive results. Indeed, using MS/MS, potential (negatively charged) suspect compounds demonstrating too high EFs of ≥ 140 could be falsified (see Fig. 7, Panels 3b and c).

**Table 2:** List of suspect compounds in ESI+ and ESI− obtained after analyzing Fraction **F**_**4**_ (sample N2) by RPLC-NTS. Three analytes were detected in both ESI polarities in separate runs. Column “MS/MS conf.” describes the confirmation by MS/MS (n – no, y – yes, and o – no MS/MS spectra recorded). The strongest acidic and basic pK_a_ values, charge number, and log *P* and log *D* values at pH 10 (log *D*_pH 10_) were simulated by Chemicalize provided by ChemAxon (11/02/2021). Analytes are sorted according to their charge number at pH 10. The last three columns list the EFs (peak heights) for Fraction **F**_**4**_ vs. the original sample N2 in ESI+ and ESI− mode as well as the peak number in the EICs shown in Fig. 6

MS polarity	Suspect analyte	MS/MS conf.	RT in min	Strong. acidic pK_a_	Strong. basic pK_a_	Charge number at pH 10	Log *P*	Log *D*_pH 10_	EF for peak height ratio **F**_**4**_/sample ESI+	EF for peak height ratio **F**_**4**_/sample ESI*−*	Peak N° Fig. 6
ESI+	Genistein	n	9.1	6.6		−3.0	3.1	−1.8	140.3		
	Candesartan	y	10.0	3.5	1.5	−2.0	5.3	0.2	1.5		1
	Ciclopirox	y	5.2	6.8		−1.0	2.2	−0.1	11.3		
	Dimethachlor CGA 354742 (dimethachlor ESA)	o	7.0	−0.8		−1.0	1.2	−1.1	70.3		
	5-methyl-1-*H*-benzotriazol; 4-methylbenzotriazole	y	7.4	9.1	0.5	−0.9	1.8	0.8	3.8		2
	3-phenylphenol; 4-phenylphenol	n	11.0	9.9		−0.6	3.3	3.0	249.2		
	rac *N,O*-didesmethyl venlafaxine	n	6.0	10.3	9.7	−0.2	1.7	1.7	5.8		
	Diphenylamine	y	13.3		0.8	0.0	3.4	3.4	7.1		3
	*N*-ethylaniline	y	5.6		4.9	0.0	1.8	1.8	270.9		
	1,3-diisopropylurea	n	6.9	15.7	−1.3	0.0	0.6	0.6	32.6		
	Levetiracetam	n	5.5		−1.6	0.0	−0.6	−0.6	227.5		
	Benzoguanamine	y	5.8	15.6	7.0	0.0	1.8	1.8	17.5		
	3-[*N*-n-butyl *N*-acetyl]aminopropionic acid-ethyl ester	n	9.7		−1.3	0.0	1.0	1.0	70.5		
	Venlafaxine	y	7.1	14.4	8.9	0.0	2.7	2.7	0.9		4
ESI+/ESI*−*	Valsartan acid	y	8.1	4.0	−1.5	−2.0	3.2	−2	1.2	1.4	5
	2-hydroxybenzothiazole	y	8.2	11.3	−1.3	−1.0	2.5	0.9	7.6	8.4	6
	L-phenylalanine	y	5.3	2.5	9.5	−0.8	−1.2	−1.8	8.6	49.7	
ESI*−*	Adipic acid	y	5.6	3.9		−2.0	0.5	−6.6		15.2	7
	1*H-*benzotriazole	y	6.4	9.0	0.2	−1.0	1.3	0.1		0.6	
	3-hydroxybenzaldehyde; 4-hydroxybenzaldehyde	y	6.7	7.3		−1.0	1.4	−0.7		3.7	
	p-toluene sulfonic acid	y	5.7	−2.1		−1.0	1.7	−0.7		2.3	
	Benzothiazole sulfonic acid	y	6.2	−2.8		−1.0	−0.3	−0.4		3.1	
	3,5-di-tert butyl salicylic acid	y	15.9	2.8		−1.0	5.1	1.5		3.5	8
	Coumatetralyl	y	12.5	5.6		−1.0	3.8	1.0		3.3	9
	Difenoxuron	o	10.2	14.0		0.0	2.7	2.7		2.3	
	Dimefuron	n	11.1	12.9		0.0	3.4	3.4		3.4

Seventy percent of the suspects listed in Table 2 have a (simulated) negative charge number (≤ −0.2) at pH 10, which was defined as the threshold for an effective FSE stacking. Thirteen of these suspects were verified by MS/MS, e.g., valsartan acid and candesartan (see Fig. 7, Panels 1a-c and 2a-cand Figure legend).

Four neutral suspects (diphenylamine, *N*-ethylaniline, benzoguanamine, and venlafaxine, see (Table 2) were verified using MS/MS. The ratio of 0.9 for the uncharged suspect venlafaxine corresponds well with the FSE mechanism: neutral analytes can reach Fraction **F**_**4**_ when diffusion takes place, broadening the injection zone. Their concentration is reduced compared to the original sample by a factor of approx. 4 (sample flow rate of 12.7 ml/min vs. fraction collection rate of 3.4 ml/min). *N*-ethylaniline had a too high EF, so a false-positive result is very likely. Other neutral suspects with too high enrichment factors were also classified as false-positives using MS/MS (e.g., levetiracetam and dimefuron). Still, some analytes (benzoguanamine and adipic acid) revealed too high peak height ratios, possibly coelution with isobaric compounds occurred. Overall, the increased selectivity by charge strongly aided in suspect screening. FSE was able to capture transformation products, e.g., the suspect valsartan acid (N°5 in Fig. 6, Table 2), a transformation product of valsartan. The non-target screening also showed that analyte polarity was not decisive for an analyte to be covered by FSE: the suspects covered a log D_pH 10_ range from −6.6 (adipic acid) to 1.5 (3,5-di-tert butylsalicylic acid). Different substance classes were covered.

## Conclusion and outlook

A new sample preparation method for the analysis of ionizable micropollutants in surface waters based on free-flow electrophoresis (FFE) in the mode field-step-electrophoresis (FSE) was presented. FSE efficiently focused acidic analytes with pK_a_ values of up to 10. It was well compatible with downstream analysis by RPLC- and HILIC-MS with very good repeatability. The comparison of its performance with established sample preparation techniques for water analysis like SPE and EC demonstrated the high potential of FSE for the analysis of ionic and ionizable micropollutants in river water and showed its broad coverage as well as its capability to increase S/N ratios by a factor of up to 6. The orthogonality of the FSE and LC separation proved advantageous as the intensity of river matrix components eluting in the void volume and matrix effects were strongly reduced especially in negative ESI polarity. A proof of concept for non-target screening was presented resulting in 17 suspects fully identified by retention time and MS/MS spectra in the relevant FSE fraction with only very minor interferences of the FSE electrolytes with the subsequent non-target screening. The additional selectivity criterion of charge by FSE proved to be useful in excluding false-positive results and thus facilitated suspect screening.

Only minor analyte losses occurred in contrast to SPE, where some polar analytes were not well retained. In contrast to direct evaporative concentration of environmental samples, FSE fractionation removed critical salt matrix components. FSE may be applied as a sample cleanup prior to EC as the enrichment would then occur after salt removal with high enrichment factors but lowered matrix effects. FSE and EC may advantageously be combined to reach both removal of inorganic salts and intense preconcentration of target analytes. An interesting future application is the analysis of marine samples with their very high salinity.

FSE was shown for the first time to be an interesting tool for screening applications in environmental analysis, especially when bearing in mind that transformation products in the environment often have a higher acidity than their parent compounds. Tuning flow vs. separation velocity in FSE will be further improved in future investigations in order to increase loadability: five times higher sample flow rates can be applied to achieve higher preconcentration during fractionation. Further enrichment can be reached by narrowing down the fractions after FSE separation, which is feasible as the FSE electrolytes chosen are volatile. FSE sample preparation methods for basic analytes have to be developed.

## Supplementary Information

Below is the link to the electronic supplementary material.

Supplementary file1 (DOCX 50 KB)

## Data Availability

Not applicable.
