# Supplementary material for: Performance of free-flow field-step electrophoresis as cleanup step for the non-target analysis of environmental water samples
Source: Anal Bioanal Chem. 2022 Jan 31;414(6):2189–204. doi: 10.1007/s00216-021-03856-w (PMC8821473; doi:10.1007/s00216-021-03856-w)
Supplement: Supplementary file 1 — Supplementary file1 (DOCX 50 KB) [file 216_2021_3856_MOESM1_ESM.docx]

Supporting Information

to

**Performance of free flow field-step-electrophoresis as clean-up step for the non-target analysis of environmental water samples**

^1^ Tobias Rösch, ^2^ Gerhard Weber, ^3^ Tobias Bader, ^1,4^ Jorina Wicht, ^1^Carolin Huhn

^1^ Institute for Physical and Theoretical Chemistry, University of Tübingen, Auf der Morgenstelle 18, Tübingen, Germany, ^2^ FFE Service GmbH, Feldkirchen, Germany

^3^Zweckverband Landeswasserversorgung, Laboratory for Operation Control and Research, Langenau, Germany, ^4^ current address: Bayerisches Landesamt für Umwelt, Augsburg, Germany

This supporting information summarizes information on sample collection and on investigations on the compatibility of field step electrophoresis with the downstream separation techniques RPLC- and HILIC-MS.

## S1. Sample collection and spiking

Blank FSE-fractions (FSE experiment using H_2_O LC-MS grade) were spiked to estimate LODs and matrix effects. Fraction 1 (**F_1_**) and combined fractions 3-5 (**F_3-5_**, see Figure S 1) were evaporated to dryness under a gentle stream of nitrogen. Reconstitution was conducted using H_2_O (RPLC) or MeCN (HILIC)

To broaden the view on possible matrix effects from FSE media, two FSE-fractions from two additional FSE experiments conducted at pH 5 and 8 (**F_pH 5_**, **F_pH 8_** containing *N*-morpholine and acetic acid at different concentrations) were prepared and spiked in the same way. Spiking concentrations were adapted to their linear range for the technique chosen for downstream analysis technique : HILIC-MS: 10, 100, 500 and 1 000 ng/l and RPLC-MS: 100, 1 000, 5 000 and 10 000 ng/l to determine LODs. Matrix effects were determined by comparing peak areas of the analytes in the FSE fraction vs. the peak areas obtained from the analysis of an aqueous standard. Concentrations were 500, 1 000 and 10 000 ng/l for HILIC-MS and RPLC-MS.

## S2. Compatibility with common subsequent separation techniques

In the following paragraph, the compatibility (matrix effects, LODs and also orthogonality to the FSE separation) with three different separation techniques was investigated, namely RPLC-MS and HILIC-MS. Details on each separation method are provided in Section 2.4 in the main text. A direct injection of fractions was possible in all cases but some disturbances and instabilities during the measurements were observed. Thus, as the FSE-media were made from volatile compounds, evaporation and reconstitution as described in Figure 1 of the main text (steps 5&6) were implemented as a general sample preparation step.

For compatibility studies, the blank samples F1, F3-5 and F_pH 5_ and F_pH 8_ (see Section S1) were used. Figure S 1A and B show the average matrix effects and LODs for all analytes obtained from the analysis of the aqueous standard and the spiked FSE fractions. Average matrix effects ranged from 80 to 110% for all three separation techniques. Lowest LODs were obtained for separations using HILIC (0.10-0.25 µg/l). Spiked samples analyzed with RPLC (C18) exhibited LODs in the low µg/l range (0.6-0.8 µg/l).


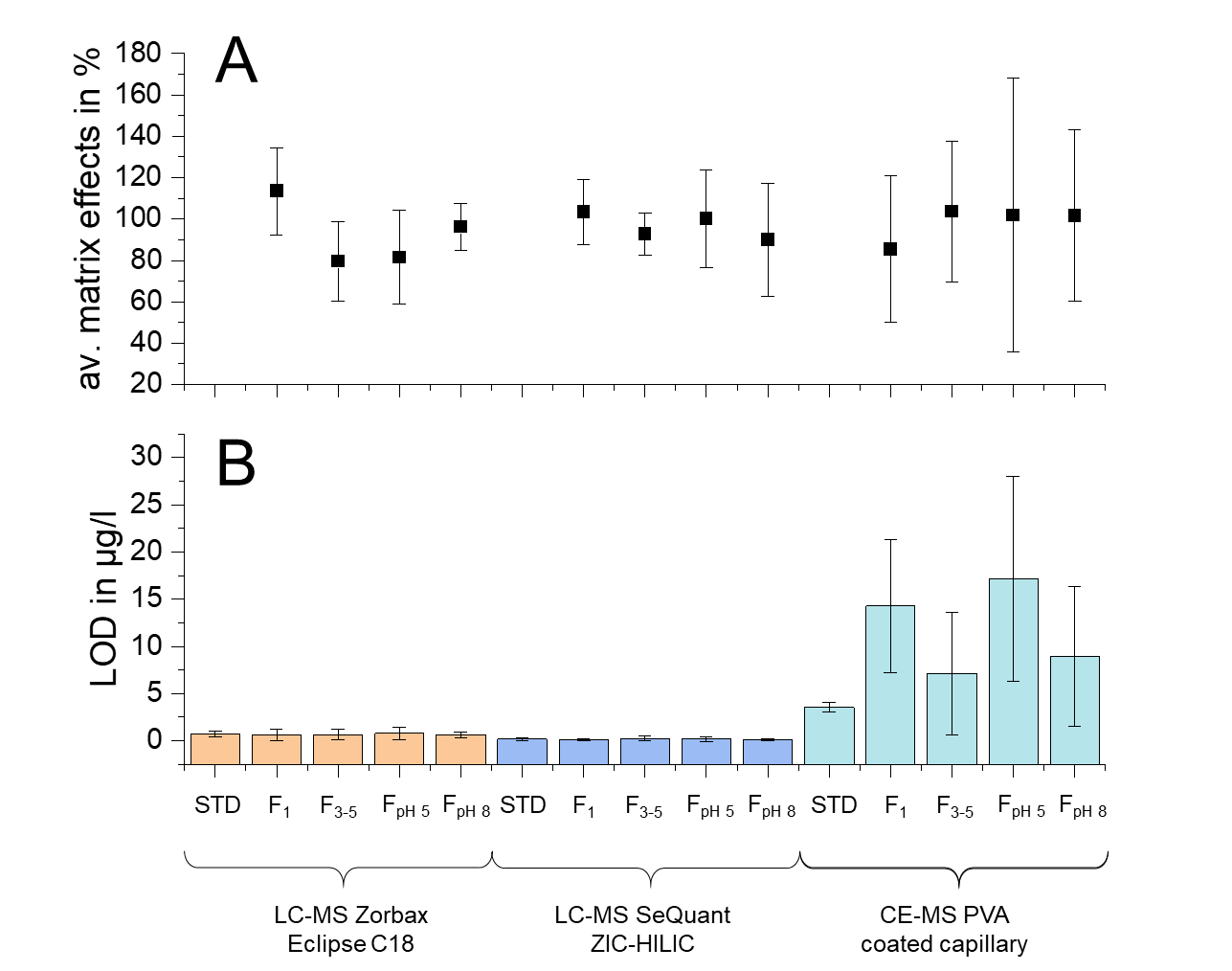


Figure S1: A: Average matrix effects for all analytes detectable in four different FSE-fractions (F_1_: fraction 1, F_3-5_: combined fractions 3-5, and fraction from an additional FSE experiments conducted at different pH: F_pH 5_: fraction containing N-morpholine and acetate at pH 5 and F_pH 8_: fraction containing N-morpholine and acetate at pH 8) determined by RPLC-MS and HILIC-MS. Determination was conducted via the recovery of analytes when spiking each fraction at a concentration of 1 000 ng/l for RPLC-MS (500 ng/l HILIC-MS) compared to the recovery using aqueous standards (for HILIC: MeCN). B: Average LODs for the model analytes obtained by the analysis of spiked FSE fractions.
